# Body Microbiota and Its Relationship With Benign and Malignant Breast Tumors: A Systematic Review

**DOI:** 10.7759/cureus.25473

**Published:** 2022-05-30

**Authors:** Ali A Samkari, Meaad Alsulami, Linah Bataweel, Rozan Altaifi, Ahmed Altaifi, Abdulaziz M Saleem, Ali H Farsi, Omar Iskanderani, Nouf Y Akeel, Nadim H Malibary, Mai S Kadi, Emad Fallatah, Mahmoud Fakiha, Alaa A Shabkah, Nora H Trabulsi

**Affiliations:** 1 Department of Surgery, King Abdulaziz University Faculty of Medicine, Jeddah, SAU; 2 Faculty of Medicine, King Abdulaziz University, Jeddah, SAU; 3 Department of Radiation Oncology, King Abdulaziz University Faculty of Medicine, Jeddah, SAU; 4 Visceral and General Surgery, Hautepierre Hospital, Strasbourg, FRA; 5 Department of Community Medicine, King Abdulaziz University Faculty of Medicine, Jeddah, SAU; 6 Department of Surgery, University of Jeddah Faculty of Medicine, Jeddah, SAU; 7 Department of Surgery, International Medical Center, Jeddah, SAU

**Keywords:** breast disease, microbial dysbiosis, microbiome, breast tissue, breast cancer

## Abstract

Breast cancer is the most frequent type of cancer as well as one of the main causes of cancer-related mortality in women. Human microbial dysbiosis, which has been related to a range of malignancies, is one of the variables that may impact the chance of developing breast disorders. In this review, we aimed to investigate the relationship between breast cancer and benign breast tumors with dysbiosis of the microbiome at different body sites. We performed a systematic review of MEDLINE, Scopus, Ovid, and Cochrane Library to identify original articles published until July 2020 that reported studies of breast disease and microbiota. Twenty-four original articles were included in the study, which looked at the features and changes in breast, gut, urine, lymph node, and sputum microbial diversity in patients with benign and malignant breast tumors. In breast cancer, the breast tissue microbiome demonstrated changes in terms of bacterial load and diversity; in benign breast tumors, the microbiome was more similar to a malignant tumor than to normal breast tissue. Triple-negative (TNBC) and triple-positive (TPBC) types of breast cancer have a distinct microbial pattern. Moreover, in breast cancer, gut microbiota displayed changes in the compositional abundance of some bacterial families and microbial metabolites synthesis. Our review concludes that breast carcinogenesis seems to be associated with microbial dysbiosis. This information can be further explored in larger-scale studies to guide new prophylactic, diagnostic, and therapeutic measures for breast cancer.

## Introduction and background

Breast cancer is the most frequent type of cancer in women and is one of the main causes of cancer-related mortality in women [1,2]. Older age, prolonged exposure to female hormones, BRCA1 and BRCA2 genes mutations, and the presence of a personal or family history of breast and other cancers are all well-known risk factors for developing breast illnesses [3,4].

One of the factors that might influence the risk of the development of breast diseases is human microbial dysbiosis [5,6]. The microbiome, as defined by Lederberg and McCray, is the ecological community of commensal, symbiotic, and pathogenic microorganisms that share our body spaces [7]. In 1960, it was difficult to understand the role of microbiota. Our understanding of microbiota has improved as genome-analyzing tools for complex microorganisms have advanced, but we still do not know much about its clinical significance [8].

Microbial dysbiosis results when maladaptation or abnormal composition occurs within the microbial community of a given organ or tissue [9]. The literature has reported a link between microbial dysbiosis and the development of a variety of cancers [10-12]. The microbiota have also been shown to help increase drug efficacy, decrease drug toxicity, and prevent cancer [13]. Other studies have concluded that microbiota could be used in the diagnosis, prediction of risk and course, and prevention of disease [14].

The association between different types of microbiota and gastrointestinal pathological conditions has been well studied. Some investigators propose an association between colorectal cancer and certain microbiota detected by fecal and oral swabs [15]. In a new field of research, recent studies have suggested an association between inflammatory bowel diseases and microbiota [16,17]. Research on different breast pathological conditions and their links to microbiota that inhabit breast tissue and other organs is limited. In this comprehensive review, we, therefore, aimed to study the characteristics and changes (dysbiosis) in breast, gut, and other body site microbiomes in relation to breast cancer and benign breast tumors.

## Review

Study protocol and registration

The study protocol is available at https://www.crd.york.ac.uk/prospero/display_record.php?RecordID=187358. The registration code is CRD42020187358.

Search strategy

From May 20 to June 3, 2020, we performed a comprehensive search of databases (MEDLINE, Cochrane Library, Ovid, and Scopus) and retrieved the literature published to June 2020, as well as the relevant reference lists of research discovered through an electronic search. The following were the MEDLINE database search terms: ("bacteria"[mesh] OR "viruses"[mesh] OR "fungi"[mesh] OR "archaea"[mesh] OR "Microbiota"[mesh]) AND ("breast diseases"[mesh] OR "breast"[mesh]). We focused our search on studies on adults and humans.

Study selection

The primary screening of the studies was done by two authors based on the title and abstract by using the search terms described above independently, and duplicate studies were removed. A full-text review was undertaken independently. When the inclusion and exclusion criteria were not met during the initial review, the articles were discarded. When uncertainty existed, a third senior author resolved the disagreement. We included all studies that matched all of the following criteria.

Study Design

We included retrospective cohort studies and secondary analyses in which the main purpose was to evaluate the microbial diversity and characteristics of breast or gastrointestinal tissue or any other body site in patients with breast cancer or benign breast tumors.

Study Subjects

Studies included adult females, 18 years and over, with breast malignancy or benign breast tumors.

Outcomes

The outcomes were the characteristics and changes (dysbiosis) in breast, gut, and other body microbiomes in relation to benign and malignant breast tumors.

Studies considered by the authors to be unrelated to the subject, non-human studies, and non-English studies were excluded.

Breast microbiota

**Figure 1:**
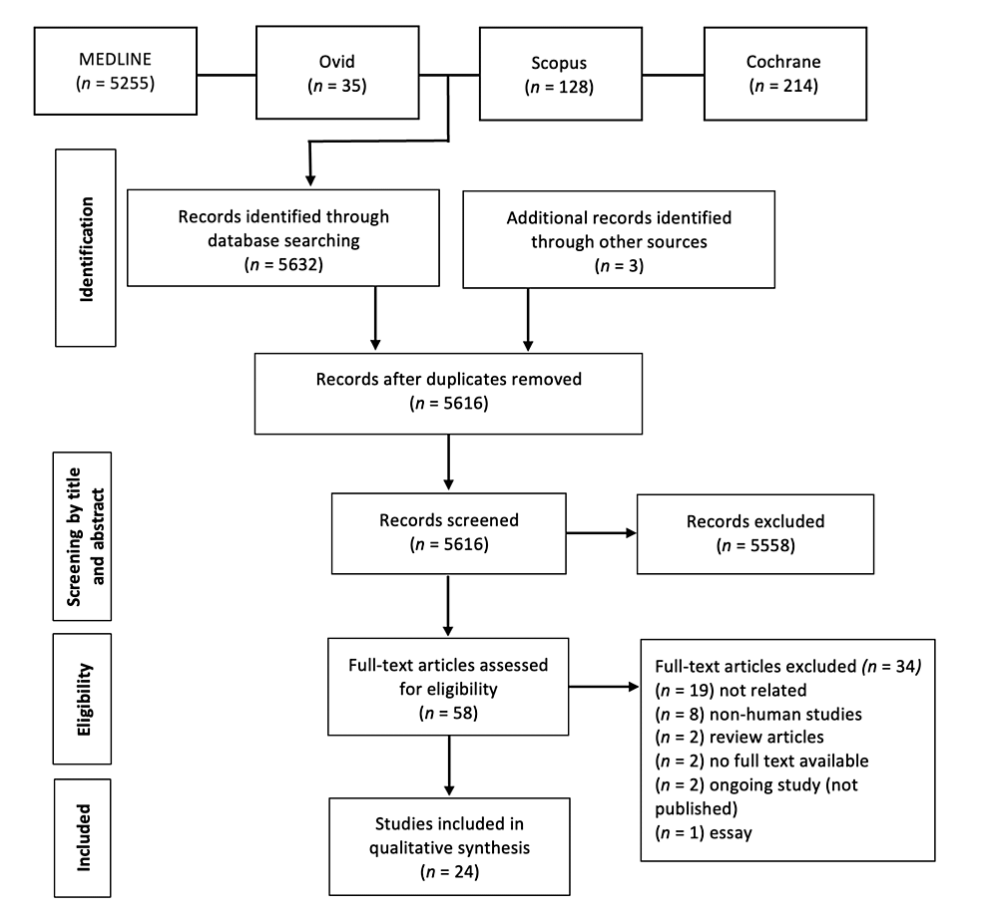
Preferred Reporting Items for Systematic Reviews and Meta-Analyses (PRISMA) flow diagram.

A total of 5616 articles were screened after the initial search, cross-referencing, and removal of duplicate studies. Inclusion and exclusion criteria were applied by reading the title and abstract. Following this step, 58 studies were included in the full-text reading. After a full-text review, 34 more studies were excluded, and a final 24 studies were selected and included in this review (Figure 1). All included studies and their main characteristics and findings are summarized in Table 1.

**Table 1:** Studies that examined microbiota and its association with benign and malignant breast tumors. AM: Akkermansia muciniphila, BaiH: bile acid induction 7α/β-hydroxysteroid dehydroxylase, BIA-ALCL: breast implant-associated anaplastic large cell lymphoma, BMI: body mass index, ER: estrogen receptor, HAM: high Akkermansia muciniphila, IgA: immunoglobulin A, LAM: low Akkermansia muciniphila, LCA: lithocholic acid, NAF: nipple aspirate fluid, NTM: nontuberculous mycobacterial lung disease, NTM-BCa: nontuberculous mycobacterial lung disease and breast cancer, PGEM: prostaglandin E metabolite, PUFA: polyunsaturated fatty acid, qPCR: quantitative polymerase chain reaction, RT-PCR: reverse transcription-polymerase chain reaction, TIL: tumor-infiltrating lymphocyte, TNBC: triple-negative breast cancer, TPBC: triple-positive breast cancer.

Reference	Type of study	Aim	Sample size	Sample site within the breast	Sample assessment methods	Main findings
Xuan et al. [18]	Cross-sectional	To investigate the potential role of microbiota in breast cancer	20 patients with estrogen receptor-positive breast cancer	Normal adjacent tissue and tumor tissue	16S rDNA pyrosequencing	The most numerous phyla in breast tissue are Firmicutes, Actinobacteria, Bacteroidetes, and Proteobacteria. *Sphingomonas yanoikuyae* was more prevalent in the normal surrounding tissue, while *Methylobacterium radiotolerans* were more numerous in tumor tissue. The breast cancer stage was shown to be inversely associated with bacterial burden in tumor tissue.
Urbaniak et al. [19]	Cross-sectional	To investigate the presence of microbiome within the mammary gland	Canadian samples: 11 benign, 27 cancer, 5 healthy Irish samples: 33 cancer, 5 healthy	From the patients, Samples were taken from outside the tumor marginal zone	16S rRNA sequencing and culture	Bacillus species, Micrococcus luteus, *Propionibacterium acnes*, and *Propionibacterium granulosum* were the most abundant species in the case as well as control tissue.
Goedert et al. [20]	Cross-sectional	To investigate the difference in gut microbiota among patients with breast cancer with regard to menopausal status.	48 postmenopausal patients with breast cancer, pretreatment, vs 48 control patients. Urine (without preservative) and feces	NA	16S rRNA and Fecal DNA gene sequencing	Faecalibacterium, Clostridiaceae, and Ruminococcaceae were found in higher concentrations in breast cancer patients. On the other hand, Lachnospiraceae and Dorea species were found in lower concentrations in breast cancer patients.
Banerjee et al. [21]	Case-control, cross-sectional	To discover the microbiota linked to TNBC.	100 TNBC, as well as 20 non-matched and 17 matched controls	TNBC, cancer tissue samples were collected. Matched-controls were collected from the same patient's normal neighboring tissue. Breast tissues from healthy people were used as non-matched controls.	PathoChip technology	When compared to other samples, the microbial profile observed in TNBC samples was strongly related to cancer samples.
Yazdi et al. [22]	Cross-sectional	To evaluate bacterial dysbiosis in sentinel lymph nodes from breast cancer patients	123 frozen sentinel lymph nodes from breast cancer patients were collected, as well as 123 normal neighboring breast tissue and 5 normal mastectomies.	Normal adjacent breast tissue	RT-PCR and pyrosequencing	Increased presence of *Methylobacterium radiotolerans* in sentinel lymph nodes.
Chan et al. [23]	Experimental	To characterize the microbiome present in NAF	23 healthy control women and 25 with a history of breast cancer NAF, nipple/areola skin swap	NA	16S rRNA gene sequencing	Alistipes species was present only in NAF from breast cancer, while Sphingomonadaceae was found to be more prevalent in healthy samples.
Urbaniak et al. [6]	Case-control, cross-sectional	To investigate the possible involvement of breast microbiota in the development of breast cancer	71 fresh breast tissue samples were collected from women, 13 of whom were benign, 45 were cancer, and 23 were healthy.	From women with cancer, tissue samples were taken from outside the marginal zone	16S rRNA gene sequencing	Patients with cancer have a higher compositional abundance of Bacillus, Enterobacteriaceae, Staphylococcus, Comamonadaceae, and Bacteroidaceae species. No significant difference across stages.
Hieken et al. [24]	Cross-sectional	To evaluate the role of breast microbiota in breast cancer development	33 patients: 16 benign, 17 cancer for breast tissue, buccal swap, skin swap, full-thickness skin biopsy	From women with tumors, the tissue was obtained from normal adjacent breast tissue.	16S rRNA sequencing	The microbiome of breast tissue is different from the microbiota of breast skin tissue, skin swap, and buccal swap. In malignant samples, Atopobium, Fusobacterium, Hydrogenophaga, Gluconacetobacter, and Lactobacillus genera were more abundant.
Luu et al. [25]	Cross-sectional	To investigate the association between microbiota composition and clinical and biological parameters of breast cancer patients	A stool sample from 31 with early-stage breast cancer: 23 patients had a normal body mass index (BMI), and 8 were overweight or obese	NA	Quantitative PCR (qPCR) targeting 16S rRNA	The amount of Faecalibacterium, Firmicutes, Blautia species, prausnitzii, and Eggerthella lenta bacteria was considerably lower in overweight and obese individuals compared to the normal BMI group. The number of Blautia species grew significantly with grade.
Wang et al. [26]	Case-control, cross-sectional	To explore the microbiome of breast tissue and its relationship to breast cancer	78 patients: 57 with invasive breast cancer, 21 healthy controls mid-stream clean-catch urine samples, a saline mouth rinse samples, and samples of tumor and nearby normal breast tissue were obtained.	Control breast tissue samples were taken on the right and left sides. In addition, tumor tissue and ipsilateral neighboring normal tissue were taken from patients.	DNA extraction 16S rRNA gene sequencing	Methylobacteriaceae species were significantly decreased in patients with cancer, while Alcaligenaceae species were increased in cancer samples relative to non-cancer samples. No significant difference in oral rinse microbiome between cancer patients and healthy controls. The difference in the urine microbiome was largely driven by menopausal status.
Thompson et al. [5]	Cross-sectional	To study the breast microbiota and its association with the tumor expression profile	668 breast tumor tissues 72 normal adjacent tissue	Tumor tissue and non-cancerous adjacent tissue	16S rRNA gene sequencing	Actinobacteria, Proteobacteria, and Firmicutes were the most numerous phyla in breast tissue. Actinobacteria species were plentiful in non-cancerous tissue nearby. Proteobacteria were found in greater abundance in tumor tissue. *Mycobacterium phlei* and *Mycobacterium fortuitum* were found in higher concentrations in tumor samples.
Goedert et al. [27]	Case-control, cross-sectional	To study the postmenopausal breast cancer associations with urinary levels of estrogens and estrogen metabolites, inflammation marker PGE-M, and finally, with IgA positive and IgA negative fecal microbiota	48 postmenopausal breast cancer cases and 48 postmenopausal controls Urine (without preservative) and stool samples	NA	16S rRNA gene sequencing	Alpha diversity is drastically diminished in breast cancer patients. Furthermore, the makeup of their IgA-positive and IgA-negative fecal microbiota has changed.
Mikó et al. [28]	Experimental	To investigate the association between changes in the microbiome, microbiome-derived metabolites, and breast cancer	Serum and stool samples from 56 patients and 56 healthy controls Fecal samples from 46 patients and 48 healthy controls	NA	DNA extraction from fecal samples and qPCR	Patients with early-stage breast cancer versus control women had reduced serum LCA levels, a reduced chenodeoxycholic acid to LCA ratio, and a lower abundance of BaiH of Clostridium sordellii, Staphylococcus aureus and Pseudomonas putida.
Banerjee et al. [29]	Cross-sectional	To explore the microbiome diversity among the different types of breast cancer.	50 ER positive, 34 HER2/neu positive, 24 TPBC, 40 TNBC, 20 healthy controls	Breast cancer tissues and control breast samples from healthy individuals	Pan-pathogen microarray (PathoChip) strategy	TNBC and TPBC exhibit unique microbial patterns, but ER-positive and HER2/neu-positive breast cancer samples have comparable microbial profiles.
Zhu et al. [30]	Case-control, cross-sectional	To compare the gut microbial community and its functional capabilities between patients with breast cancer and healthy controls	Fecal samples from 18 premenopausal patients with breast cancer, 25 premenopausal healthy controls 44 postmenopausal patients with breast cancer, 46 postmenopausal healthy controls	NA	DNA sequencing	Gut bacterial species composition seems to be different between postmenopausal patients and postmenopausal healthy control.
Meng et al. [31]	Cross-sectional	To examine the microbiome of breast tissue from individuals with benign and cancers of various histological grades.	22 benign, 72 patients with invasive breast cancer	Samples were taken from either benign or malignant tumor tissue	16S rRNA gene amplicon sequencing	Micrococcaceae, Propionicimonas, Rhodobacteraceae, Caulobacteraceae, Methylobacteriaceae, and Nocardioidaceae familes were found in breast tissues from patients with malignant tumors.
Costantini et al. [32]	Cross-sectional	To examine the 16S-rRNA gene for the hypervariable region that best represents the microbiome in breast tissue.	Normal and tumor tissues were obtained from 9 core needle biopsies and 6 surgical excisional biopsies.	Paired normal and tumor tissues	16S rRNA gene (V3) sequencing	Proteobacteria was the most numerous phylum among all areas, followed by Firmicutes, Actinobacteria, and Bacteroidetes.
Kovács et al. [33]	Experimental	To assess the ability of cadaverine to influence breast cancer cell behavior	48 postmenopausal patients with breast cancer, and 48 control	NA	Fecal DNA samples	DNAs from *Enterobacter cloacae*, CadA *E. coli*, and LdcC *E. coli*, were identified less often in cancer patients. In stage 0 breast cancer patients, levels of CadA and LdcC were found to be lower than their levels in other individuals. In stage 1 breast cancer patients, fecal samples showed lower levels of *E. coli* LdcC protein as compared to healthy females.
Shi et al. [34]	Cross-sectional	To assess the association between the diversity of the gastrointestinal microbiome with the patterns of expression TILs in patients with breast cancer	80 patients with breast cancer	Tumor tissues	Fresh fecal samples, 16S ribosomal RNA genes	Among different TIL expression groups in a patient with breast cancer, the gut microbiome diversity was distinct and compositionally different.
Philley et al. [35]	Cross-sectional	To identify the population of pathogenic microbes residing with the Mycobacterium avium complex species in NTM-infected women	Total of 29 samples Sputum samples from 5 healthy women, 5 women with NTM, and 15 women with both -NTM and breast cancer (NTM-BCa); sera extracellular vesicles from 4 of 15 NTM-BCa cases	NA	16S rDNA sequencing	Presence of diverse microbial community in the sputum and the extracellular vesicles in women with NTM and in women with NTM-BCa. These microbiota were dominated by Fusobacterium, Bacteroides, and Allistipes, which have estrobolome activity and are associated with breast and other type of cancers.
Walker et al. [36]	Cross-sectional	To study the difference in bacterial species colonizing the implanted breast with BIA-ALCL and those colonizing the contralateral control breast	7 patients with BIA-ALCL and contralateral controls	Specimens obtained from (implant, capsule, skin, and parenchyma)	16S rRNA microbiome sequencing and culture	No significant difference was found in Shannon and alpha diversity metrics between samples from BIA-ALCL and contralateral control.
Horigome et al. [37]	Cross-sectional	To study the association of blood PUFAs with the gastrointestinal microbiota in breast cancer survivors	The drop of capillary blood for PUFAs and fecal samples from 126 participants who had been diagnosed with invasive breast cancer over 1 year ago	NA	16S rRNA sequencing	An increased level of docosahexaenoic acid was associated with the increased relative abundance of Bifidobacterium, which belongs to the *Actinobacteria phylum*. A positive association was found between the relative abundance of Actinobacteria and Bifidobacterium and the levels of eicosapentaenoic acid.
Chiba et al. [38]	Retrospective cohort	To evaluate whether neoadjuvant chemotherapy modulates the tumor microbiome and the potential impact of microbes on breast cancer signaling	Neoadjuvant chemotherapy-treated patients (n = 15) Women with no prior therapy at the time of operation (n = 18)	Tumor tissues	Breast tissue 16S rRNA sequencing	Chemotherapy administration significantly increased breast tumor Pseudomonas spp. Primary breast tumors from patients who developed distant metastases displayed an increased tumoral abundance of Brevundimonas and Staphylococcus.
Frugé et al. [39]	Secondary analysis of pooled participants in a randomized controlled trial	To examine characteristics of overweight and obese female patients with early-stage breast cancer in relation to Akkermansia muciniphila relative abundance in the gut microbiome	32 women with stage 0 to II breast cancer, fecal samples, phlebotomy	NA	16s rRNA sequencing	In females with higher body fat, AM number was lower. Alpha diversity was higher in females with HAM. Prevotella and Lactobacillus were higher, and Clostridium, Campylobacter, and Helicobacter genera were lower in HAM vs. LAM.

Breast Tissue Microbiota

The existence of microbes in breast tissue has recently been explored. Different types of microbiota have been found that are distinct and clearly distinguishable from those found in the overlying skin tissue [18,19]. Proteobacteria were the most prevalent bacterial phyla identified in the included articles among breast tissue samples taken from benign, malignant, and healthy breasts, followed by Actinobacteria and Firmicutes [5,18,19,32].

Benign vs. Malignant vs. Healthy

The microbiota appears to have a role in both benign and malignant disorders, as the profiles of benign tumors were more similar to those of normal neighboring tissue from women with malignant tumors than to those of tissue from healthy patients [6]. Nevertheless, studies have shown some differences in microbial profiles between the two-disease status [5,19].

Normal adjacent tissues from patients with breast cancer exhibited a considerably greater number of certain bacteria, including Bacillus, Staphylococcus, and members of the Enterobacteriaceae family, as compared to patients with benign breast tumors and healthy women [6]. Moreover, proteobacteria relative abundance was found to be significantly higher in malignant disease than in benign disease [5].

Furthermore, the fluid aspirated from the nipple in patients with breast cancer (ductal carcinoma) and from healthy controls differed significantly in beta diversity [23], suggesting a possible role of bacterial dysbiosis in cancer formation.

Malignant vs. Normal Adjacent

Several studies showed that tissue from malignant tumors and normal adjacent tissue of the same breast shared similar bacterial diversity [6,26,32]. However, other studies found differences in microbial profiles in terms of the relative abundance of some bacteria. At the genus level, Xuan et al. found the bacterium *Sphingomonas yanoikuyae* was most prevalent in adjacent normal tissue, and the bacterium *Methylobacterium radiotolerans* was most prevalent in tumor tissue [12,18]. In contrast, Wang et al. [26] found that the genus Methylobacterium was considerably lower in cancer patients compared to non-cancer individuals. This difference between studies might be due to different extraction techniques or types of sample preparation.

When compared to non-cancerous surrounding samples, *Mycobacterium phlei* and *Mycobacterium fortuitum* were shown to be the most abundant species in tumor tissues [5].

On quantitative analysis, Xuan et al. found that the tissue of estrogen receptor-positive breast cancer has 10-fold more bacteria when compared to paired normal tissue from the same patients [18]. Nevertheless, despite this increase in microbial load in breast cancer tissue, antibacterial responses within cancer tissues were found to be significantly downregulated compared to normal breast tissue [12]. These data suggest a possibility that microbiota has the ability to influence the local immune microenvironment of the breast.

Role in carcinogenesis

The bacterial load in breast tissue seems to be inversely correlated with the stage of breast cancer, as the highest copy numbers of bacterial DNA were found in tumor tissues from patients with early-stage cancer, and it decreased with the more advanced stage. However, this association was not found with adjacent normal tissue from the same patients as no difference in bacterial load was found across stages [18]. These observations have significance in guiding new diagnostic implications for breast cancer.

The histological grade of breast cancer has also shown some effects on the tumor microbial profile [31]. Meng et al. discovered that when tumor grade increased, the compositional abundance of the Bacteriodaceae family was reduced. Furthermore, as malignancy grows, so does the prevalence of the genus Agrococcus [31].

Luminar type of breast cancer may have an influence on the microbial community of tumor tissue. Banerjee et al. discovered unique patterns of bacterial, viral, fungal, and parasitic profiles in triple-negative (TNBC) and triple-positive (TPBC) breast cancer samples. The microbial profiles of human epidermal growth factor receptor 2 (HER2/neu)-positive and estrogen receptor (ER)-positive samples, on the other hand, were similar [21,29]. When hormone receptor-positive breast cancer samples were compared to hormone receptor-negative samples, Wang et al. discovered a substantial drop in Methylobacterium [26]. Furthermore, samples with histopathologic evidence of lymphovascular invasion contained fewer methylobacterium than those without lymphovascular invasion.

It has not yet been fully elucidated whether there is a specific microbial species that plays a role in breast cancer development. However, *Escherichia coli* isolated from adjacent normal tissue of patients with breast cancer has been shown to generate DNA double-stranded breaks, the most harmful sort of DNA damage [6].

16S rRNA sequencing data of breast tumors from untreated patients and from those treated with neoadjuvant chemotherapy indicates that chemotherapy increases the tumor proportional abundance of Pseudomonas by 85%, suggesting that chemotherapy induces preferential growth or survival of some types of bacteria [38].

Gut microbiota

The gut microbiota also seems to undergo changes in the presence of breast cancer. The number of these species in postmenopausal patients with breast cancer is higher than that in healthy controls, with a higher abundance of specific species, including *E. coli*, *Prevotella amnii*, and *Enterococcus gallinarum* [30].

The prevalence of Blautia species in the gut microbiota increased substantially with breast cancer grade. The number of *Clostridium coccoides*, *Clostridium leptum*, and Bacteroidetes clusters was significantly higher in clinical stage II/III breast cancer than in clinical stage 0/I breast cancer [25].

In early-stage breast cancer, bacterial cadaverine biosynthesis is decreased in the gut, resulting in less production of anti-cancer bacterial metabolites [33]. Lithocholic acid (LCA), another bacterial metabolite that can slow the growth of breast cancer, was also shown to be decreased in the guts of individuals with early-stage breast cancer [28]. The level of expression of tumor‑infiltrating lymphocytes (TIL), an indicator of tumor immunity in solid cancers with prognostic value, was associated with gut microbial diversity in patients with breast cancer [34].

Obesity was linked to a considerably decreased total number of *Eggerthella lenta* bacteria, Blautia, *Faecalibacterium prausnitzii*, and Firmicutes species in early-stage breast cancer as a probable cofounder [25]. Furthermore, the proportions of Akkermansia muciniphila (AM) in the gut microbiota were lower in individuals with breast cancer who had increased body fat [39].

Microbiota of other body sites

The difference in diversity of the urinary microbiome between patients with breast cancer and healthy women was largely related to menopausal status, with peri/postmenopausal samples being significantly more diverse than premenopausal samples [26].

Oral rinse microbiomes have also been investigated. There was no significant difference between breast cancer patients and healthy women [26].

The presence of *Methylobacterium radiotolerans*, a species that was more abundant in tumor tissue than in normal paired tissue [18], was investigated in pathologically negative sentinel lymph nodes and found to be significantly associated with a higher stage of breast cancer [22].

No significant difference was observed in alpha and beta diversity in the microbiome of sputum samples in a comparison of samples from patients with nontuberculous mycobacterial lung disease with and without breast cancer [35].

Discussion

In benign and malignant breast cancer, the role of microbiota is poorly understood in the literature. Several hypotheses have been proposed, including its ability to induce chronic inflammation and regulate immunity, induce DNA damage, and modulate estrogen metabolism [40,41]. This review summarized the currently available data on the role that human microbiota plays in benign and malignant breast tumors. 

A number of characteristics of breast microbiota have been discovered in the last few years. A wide diversity of microorganisms is specific to breast tissue and distinct from the microbiota of other body sites [29,24]. The most numerous microbial phyla detected in breast tissue, according to the evaluation of these microbiomes, are Firmicutes and Proteobacteria [5,18,19,32]. However, in one study, bacteria from the phylum Bacteroidetes were the most prevalent, with very few Proteobacteria [24]. Possible explanations for the presence of a microbiome within breast tissue include the passage of skin microbiome through the nipple, during lactation or sexual contact through nipple-oral contact, and finally, translocation of the gut bacteria [36], with the data suggesting more support for the latter hypothesis [42-44].

Microbial dysbiosis, which occurs when the abundance of some species within the microbial community changes relative to others, can result in the microbial community's typical function being lost [9]. Some authors reported dysbiosis of the breast tissue microbiome in patients with breast cancer [5,6,18,21,23,29,31,32,36]. Moreover, the bacterial load also differed according to the clinical stage of breast cancer [18]. These findings demonstrate an association between dysbiosis and breast cancer. However, it is unclear whether microbial dysbiosis is merely secondary to cancer development or a causative agent in breast carcinogenesis.

We have described the potential role of some types of bacteria in carcinogenesis, including E. coli and Staphylococcus species. Both were shown to be more common in breast tissue from breast cancer patients and had the ability to produce DNA double-stranded breaks, a condition known to possibly cause cancer [6,45]. Benign breast tumors, unexpectedly, have a microbial profile similar to that of malignant tumors [6]. Urbaniak et al. [6] hypothesized that a lower level of DNA-damaging bacteria in benign tumors could be a possible factor in preventing malignant transformation.

The gut microbiota and its relationship with estrogens have been well investigated in the literature. β-glucuronidase secreted by gut bacteria allows gut microbiota to bind to estrogen receptors (ER) [10]. ER activation promotes cell proliferation, which is a well-defined process in breast cancer [46].

The studies in our review showed that the gut microbiomes display changes among breast cancer patients. Postmenopausal women with breast cancer had an altered gut microbial composition in both alpha and beta diversity metrics [20,30]. Moreover, although not statistically significant, this change was associated with a higher level of urinary estrogens in patients with breast cancer [35]. Furthermore, a significant correlation between estrogen-independency and immunoglobulin A IgA+/IgA− gut microbiota was found in postmenopausal patients with breast cancer [27]. These data suggest a relationship between gut microbiota, estrogen levels, and breast cancer.

Gut bacterial metabolites play a major role in microbiome-to-host signaling [47-50]. According to one study, the levels of LCA, one of the gut microbial metabolites, are lower in patients with breast cancer, especially in the early stages 0/I [28]. According to the same study, LCA performs an antineoplastic function in breast cancer cells. It specifically inhibits vascular endothelial growth factor production, epithelial-to-mesenchymal transition, and metastasis development. Furthermore, in animal models, it stimulates antitumor immunity and alterations in metabolism [28]. These findings are concordant with previous reports in which the same metabolite was found to be inversely correlated with Ki-67, a proliferation index in breast cancer [51].

Cadaverine is another microbial metabolite generated by the LdcC and CadA enzymes during the decarboxylation of lysine [52,53]. It exerts its effects through the trace amine-associated receptors (TAARs) TAAR1, TAAR8, and TAAR9. Previously, TAAR1 was also found to be related to inhibition of breast cancer growth and with a favorable effect on the overall survival of patients with primary breast cancer [54]. In our review, we described the results of a study by Kovács et al. [33], who showed that DNA coding for bacterial enzymes responsible for cadaverine production is decreased in the fecal microbiome of patients with early breast cancer. Moreover, prolonged survival among patients with early-stage breast cancer was associated with higher expression of lysine decarboxylase [33]. These findings suggest that gut microbial dysbiosis in patients with breast cancer leads to a a decreased production of antineoplastic bacterial metabolites.

In breast cancer, immune cell infiltration, specifically cytotoxic CD8+ T cells, has predicted an improved prognosis [55-57]. Additionally, tumor-infiltrating lymphocytes (TILs) found in breast cancer prior to chemotherapy can predict pathological complete response and improve prognosis [58]. Shi et al. [34] investigated the TILs expression in breast cancer in relation to the gut microbiome and found that microbial diversity is different among different expression levels of TILs. Higher TILs expression was associated with a higher diversity of gut microbes. Moreover, the compositional abundance of some microbiota species was different according to TILs expression levels. These findings suggest a potential role for gut microbiota in the prognosis of patients with breast cancer.

To the best of our knowledge, this is the first systematic review to examine the relationship between human microbiota and benign and malignant breast tumors. The limitations of this review are the inclusion of small retrospective studies and the heterogenicity of the results due to the use of different sampling techniques; the description of different body site microbiomes, and the study of different pathological characteristics. Further large-scale studies, including clinical trials, are needed to confirm this association and to discover the possible clinical applications of microbiota in the prevention and diagnosis of breast cancer, as well as therapeutic interventions. Furthermore, studies are required that focus more on specific members of the microbiome, such as *E. coli* and Staphylococcus, which could contribute to a better understanding of the pathophysiological characteristics of breast cancer.

## Conclusions

Current data have linked microbial dysbiosis with breast cancer. Across different histological types, stages, and grades of breast cancer, both the breast and the gut microbiome display changes. However, the exact mechanism underlying these clinical observations is poorly understood. Moreover, yet to be fully identified is whether this microbial alteration causes cancer or is one of the consequences of carcinogenesis, and whether there are specific microbial agents that contribute to the pathophysiological characteristics of this disease.
